# Contemporary strategies for donor heart preservation in heart transplantation

**DOI:** 10.3389/frtra.2026.1825471

**Published:** 2026-07-02

**Authors:** Joseph Israeli, Steven Pham, Jennie Ngai

**Affiliations:** 1 NYU Langone Health, New York, NY, United States; 2 Department of Anesthesiology, Perioperative Care and Pain Medicine, NYU Grossman School of Medicine, New York, NY, United States

**Keywords:** donor heart preservation, donor organ preservation, heart transplantation, ischemia-reperfusion injury, machine perfusion, organ preservation solution

## Abstract

Heart transplantation outcomes have tremendously improved over the nearly fifty years that the procedure has been performed. As demand continues to grow, there is a persistent shortage of organs. Compounding this is the potential for organs to deteriorate with increasing preservation and transport times. New technologies have been developed to address these issues. These include novel physiologic preservation solutions and preservation devices (which range from those that optimize temperature control to those that use machine perfusion or combinations of these facets) to minimize the risk of ischemic injury. Research is ongoing into these techniques and their potential to improve outcomes by protecting donor organs (particularly in cases of prolonged ischemic times) and expand the donor pool by better preserving grafts from marginal and/or donation after circulatory death donors. There is even research into utilizing these technologies to enable safe cardiac xenograft implantation as well as their role in beating heart transplantation.

## Introduction

Since the first successful human-to-human heart transplant in 1967 resulted in 18 days of post-transplant survival (with the patient succumbing to severe pneumonia while on heavy immunosuppressants) (1), the field of cardiac transplantation has undergone substantial evolution. During the seminal operation, the donor and recipient were in adjacent operating rooms, so that the heart could be transplanted immediately from a donation after circulatory death (DCD) donor (2). Topical hypothermia was used by immersing the graft in iced saline for the brief interim after removal (3). This approach of performing the procurement and transplantation procedures simultaneously in adjacent operating rooms continued until the establishment of brain death criteria in the 1970s. As distance procurement expanded the availability of this surgery, focus shifted to graft preservation largely through cardioplegic arrest and static cold storage (2). By 2022, median post-transplant survival approached 12 years, with conditional survival for those surviving the first year nearing 15 years. This represents approximately 10 additional life years gained compared with the median survival of under 2 years for patients with stage D heart failure (4). Despite these advances, the fundamental limitation of heart transplantation has remained unchanged: demand continues to far exceed supply. This imbalance has intensified as an aging population survives longer than ever before with advanced heart disease. A 2015 JAMA editorial (5) estimated that about 100,000 to 200,000 adults in the United States were both sufficiently ill to require transplantation and healthy enough to benefit; however, the organ procurement and transplantation network/scientific registry of transplant recipients (OPTN/SRTR) 2023 registry revealed that only 4,000–4,600 transplants are performed annually in the US (6). There are approximately 3,500–4,000 adults actively waiting for a new heart at any given time. The waitlist is continuously growing (6) as new patients are added and demand is consistently and increasingly unmet amongst existing patients (about 2,700–3,700 patients historically carry over year to year, representing those who did not match for a transplant) (5). The number of patients on the waitlist does not include patients delisted resultant of deterioration, expiration, or never meeting criteria for heart transplant listing. The discrepancy between those in need of heart transplant and those listed may reflect multifactorial components including referral patterns, geographic access to transplant centers, strict eligibility criteria (which may further exacerbate racial healthcare disparities), and psychosocial screening regulations (7). To mitigate these limitations, contemporary efforts are focused on using extended donor criteria to expand the supply of transplant organs, extending recipient criteria, optimizing organ allocation, and enhancing organ preservation to reduce graft failure rates. We will focus on recently developed methods and technologies that aim to improve organ preservation and increase the number of potential heart transplant recipients.

## Fundamental concepts and issues

Effective donor organ preservation and transportation are central determinants of allograft viability and early post-transplant outcomes. Primary graft dysfunction (PGD), a form of graft failure, is the leading cause of early mortality following heart transplantation and occurs in 7%–10% of recipients. PGD occurs within the first 24–72 h post-transplant in the absence of hyperacute rejection or surgical factors (8). Common signs include cardiogenic shock with cardiac index less than 2 L/min and/or LVEF under 40%, hypotension requiring vasoactive support, and elevated filling pressures (8). It may affect either the left, right, or both ventricles (most common subtype) (9). Requirement of inotrope or MCS determines PGD grade as mild, moderate, or severe (10). PGD may also contribute to tricuspid regurgitation (TR), which is the most common valvular abnormality following heart transplantation. TR is frequently associated with right ventricular dysfunction in the early post-transplant period. The etiology is often secondary due to annular dilation, and it may improve with recovery from PGD (11–14). Although tachycardia may be seen in shock secondary to PGD, it is important to recall that the transplanted heart is denervated and has chronotropic incompetence, so the tachycardic response to shock may be blunted or non-existent (these hearts commonly compensate through an increase in stroke volume) (15). Mixed venous oxygen saturation and lactate measurements also tend to increase (16). PGD represents the clinical manifestation of ischemia reperfusion injury (IRI) incurred during storage and transplantation (9, 17). IRI arises from a complex interplay between progressive ischemic injury during organ storage and paradoxical exacerbation of cellular damage upon reperfusion (18). Preservation modality, solution composition, and transport logistics are critical variables that must be optimized to minimize IRI and preserve both myocardial viability and endothelial integrity.

**Figure 1:**
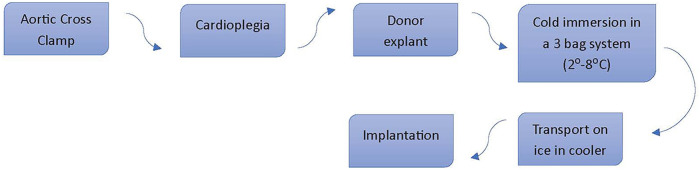
SCS—process from donation to transplant.

Static cold storage (SCS) remains the global standard for donor heart preservation. The heart is flushed with a cold preservation solution (2 to 8 degrees C) and transported in a container with ice. Please see Figure 1 for a flowchart of the SCS donor heart preservation process. Hypothermia significantly reduces myocardial metabolic demand; however, SCS provides neither oxygen nor metabolic substrates, resulting in ongoing anaerobic metabolism and time-dependent ischemic injury. As a result, the risk of IRI increases proportionally to cold ischemia time (CIT), making transport duration and logistical efficiency key determinants of graft quality (19).

Large registry analyses have demonstrated that prolonged ischemia time is independently associated with increased early mortality and PGD rates. Data derived from the United Network for Organ Sharing (UNOS) database, encompassing over 36,000 adult heart transplants, have shown a statistically significant increase in mortality once CIT exceeds approximately three hours, with each additional hour conferring a progressively higher hazard ratio (20). Recipient outcomes following SCS-preserved grafts are associated with varying PGD rates. A recent single center trial cited these at 29% overall PGD. However, it found severe PGD rates of 20% which correlate with a markedly increased risk of one-year mortality (19). Although one-year survival in adult recipients remains high (approximately 90%–97%), the burden of early graft dysfunction and subsequent morbidity/mortality as a function of increasing storage times with longer travel distances continues to represent a major limitation of SCS-based preservation strategies (21, 22). The distance limitation inherent to SCS restricts its utility in broadening access to organs among matched recipients.

CIT is tolerated longer in pediatric heart transplant recipients than adult recipients. A retrospective study of 363 pediatric recipients found no significant difference in graft survival, rejection episodes, or hospital readmissions when comparing donor hearts with CIT over 8 h and those under 90 min. The authors further supported these findings with five years of follow up that showed similar rates of post-transplant CAD and graft loss (23). A UNOS database study further affirmed these findings by suggesting that graft failure rates in patients under 18 years old do not differ significantly between various CIT categories (under 4 h, 4–6 h, over 6 h) (24). The suggestion is that increased CIT (using single dose cold crystalloid cardioplegia solution) does not increase deleterious outcomes in pediatric patients, unlike its impact in adult patients. Outcomes in pediatric patients show improvements in long term survival over successive eras, and survival has routinely exceeded 15 years in a 50-year analysis of 567 pediatric patients (25).

## State of the art

Advances in donor heart preservation have increasingly focused on mitigating IRI. The ideal temperature for organ preservation has theoretically been shown to be from 4 to 8 degrees Celsius (26), temperatures that are either too high or low can cause damage. SCS encompasses the inherent shortcoming of incomplete temperature control. The SCS “three bag system” simply includes the organ within a first bag (where it is immersed in preservation solution) that is then placed in a second bag filled with saline, which is then placed within a third bag containing saline, and then into an ice box. This results in non-homogenous cooling of the graft with potentially discrepant temperatures between different parts of the organ.

Alternative preservation modalities aim to extend safe transport durations, expand geographic sharing, and improve utilization of extended criteria donor hearts (27). Research suggests that when advanced preservation strategies (particularly machine perfusion) are employed, extended transport distances exceeding 500 miles can be achieved for DBD donors with post-transplant outcomes comparable to DBD standard distance (under 500 miles) donor transfers undergoing traditional SCS. In one propensity matched analysis of approximately 11,000 transplants, one-year survival and freedom from graft failure were similar between standard-distance and extended-distance groups, despite significantly longer transport times in the latter, where machine perfusion was more frequently used (28).

Alternative preservation modalities attenuate time-dependent ischemic injury through improved temperature control, oxygen delivery, or active metabolic support (26). In contrast to SCS, certain alternative preservation systems implement enhanced temperature control throughout the entire graft, further optimizing the principle of hypothermic metabolic suppression while mitigating the risks of freezing damage denaturing proteins or incomplete hypothermia ineffectively suppressing metabolism. Other contemporary systems may actively perfuse the graft. These techniques on their own or in combination allow preservation intervals that significantly and consistently exceed that of SCS (successful transplantations after out of body times of 9–10 h on advanced preservation has been reported) (19, 29, 30).

## Controlled hypothermic storage: SherpaPak cardiac transport system

**Figure 2:**
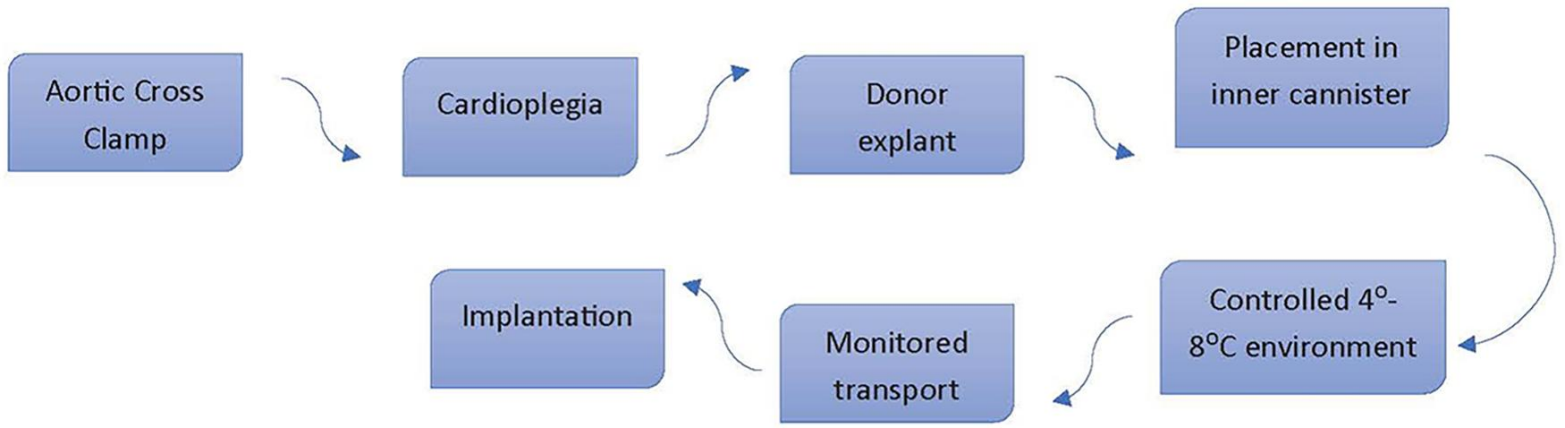
SCTS—process from donation to transplant.

The SherpaPak^TM^ Cardiac Transport System (Paragonix Technologies, Waltham, MA, USA) (SCTS) is a controlled hypothermic device that maintains the donor heart between 4 and 8 degrees Celsius. This tight temperature control mitigates the risks of freezing injury and temperature variability that SCS poses (27). Please see Figure 2 for a flowchart of the SCTS donor heart preservation process. The donor heart can be sustained within the SCTS temperature range for up to 30 h (26). To date, clinical use has involved much shorter durations. The longest reported ischemia time using this device was 330 min (5.5 h) which resulted in excellent graft function without evidence of rejection at 3 months post-transplant. Of the 330 min, 283 of those were when the graft was within the storage system (31). Thus, although it may have the potential to significantly exceed this amount of storage time, current clinical experience seems to support SCTS use for the first 5–6 h of preservation time.

SCTS may also help improve the rate of recovery from IRI. A single center study of 90 early post-transplant endomyocardial biopsies (EMB) demonstrated more rapid recovery from IRI in the early post-operative period among SCTS-preserved grafts (despite longer ischemic times) compared with SCS preserved grafts. Histopathologic analysis demonstrated a 59.3% reduction in coagulative myocyte necrosis in these specimens when using SCTS vs. SCS. This suggests that SCTS may attenuate IRI relative to conventional SCS. The investigators also reported similar rejection rates and *de novo* antibody formation, suggesting that the advanced preservation did not confer additional immunologic risk (32). Another study further assessed EMB and observed significantly lower rates of moderate to severe PGD among SCTS recipients. Between 30 recipients of SCS and 36 SCTS recipients, they found a significantly lower risk of moderate to severe PGD (11%) in SCTS recipients, vs. SCS (37%) despite higher overall risk profiles in the SCTS group, including a higher rate of bridging to transplant with a long-term ventricular assist device and higher median IMPACT scores (33).

Real-world outcomes from the GUARDIAN-Heart Registry (the largest observational dataset focused on cardiac preservation) support these findings. Among over 1,261 adult heart transplants performed in the United States between 2015 and 2024, SCTS use was associated with significantly lower rates of severe PGD (6.8% and 10.8%, respectively; *p* = 0.015) and right ventricular dysfunction (6.1% vs. 9.9%, respectively; *p* = 0.022) compared with SCS. Propensity matching further demonstrated improved two-year mortality and survival probability in SCTS recipients. Benefits persisted in organs with extended donor criteria and prolonged ischemic times. These data suggest that while ischemic injury remains time dependent, it may be attenuated under controlled hypothermic conditions (34).

Limitations of SCTS include a reliance on passive preservation which precludes metabolic assessment or targeted intervention during transport (27). As with SCS, prolonged ischemia ultimately increases PGD risk, although at a slower rate (34). Device malfunction has not been reported clinically but is a theoretical risk that could result in loss of temperature control. Reduced need for graft manipulation compared with active perfusion systems may lower the risk of iatrogenic injury, yet this has not been examined in the literature. Collectively, available evidence supports SCTS as a clinically meaningful refinement of hypothermic storage, particularly in scenarios involving extended transport or extended criteria donors.

## Normothermic *ex vivo* perfusion: organ care system

**Figure 3:**
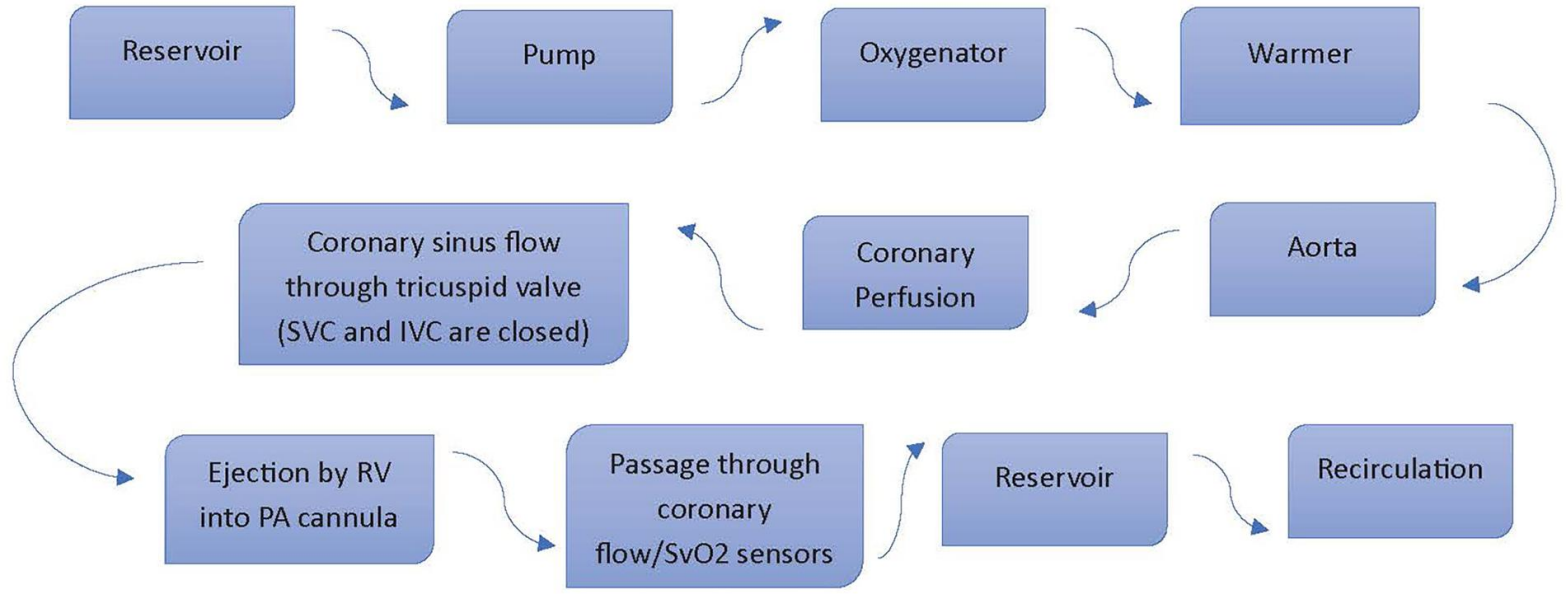
Flow through the OCS machine.

The Organ Care System^TM^ (TransMedics, Andover, MA, USA) (OCS) is a normothermic, pulsatile, *ex vivo* perfusion system designed to maintain the donor heart in a beating, metabolically active state at 34 degrees C. Continuous oxygenated perfusion aims to prevent anaerobic injury and allow continuous assessment of viability through lactate levels, coronary flow, aortic pressure, and visual inspection of contractility (35, 36). Please see Figure 3 for a schematic of flow through the OCS machine. This approach theoretically enables safer utilization of DCD, distant, and marginal grafts while reducing ischemic injury.

**Figure 4:**
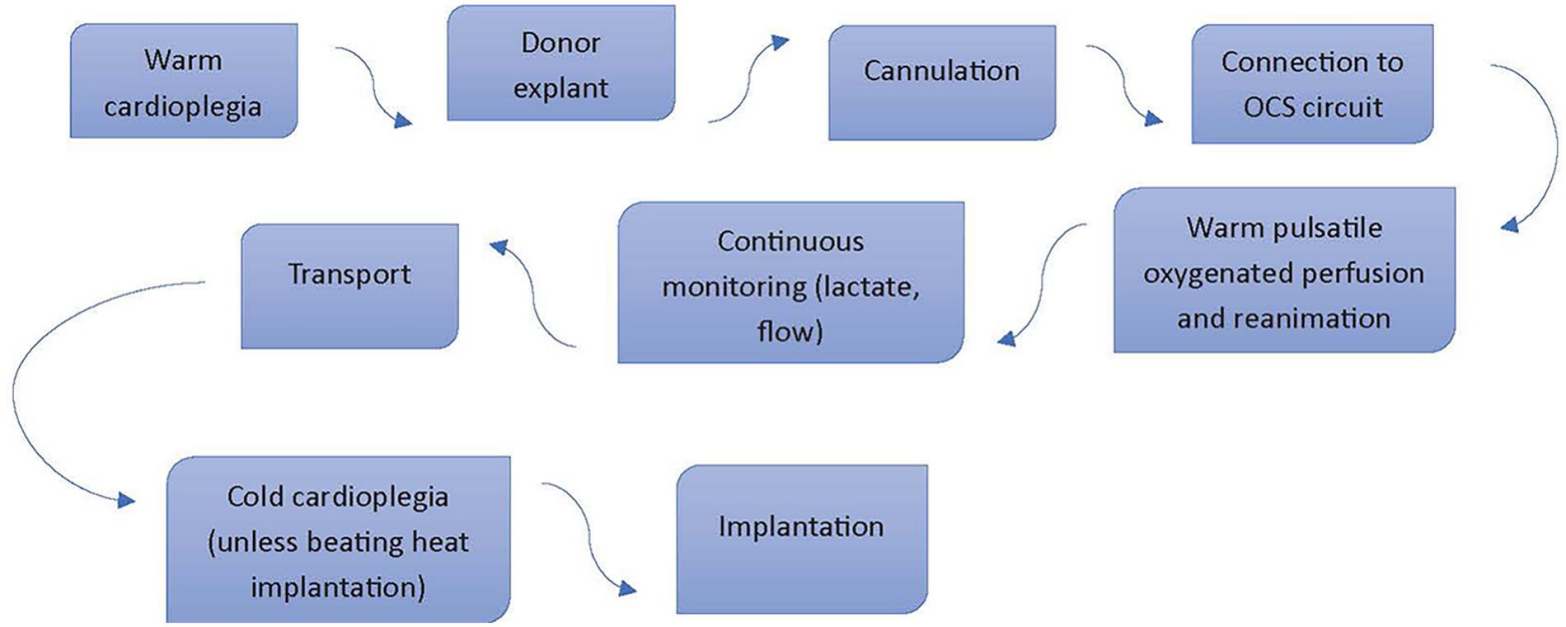
Process from donation to transplant in OCS.

OCS use is associated with increased logistical complexity, higher cost, and technical risks related to cannulation and system setup (35). Accordingly, OCS incurs costs for personnel with specialized training and dedicated organ recovery teams required to institute and maintain the mechanical circulation of the graft (37). Graft instrumentation may also increase ischemic time prior to perfusion initiation. The period following cross clamping of the donor aorta is considered CIT because the heart is normally arrested with cold cardioplegic solution and kept cold (typically with topical ice or cold saline). The hypothermia is continued until the heart is connected to the OCS and reanimated, at which point it is perfused with warm, oxygenated, nutrient enriched blood, and contractility returns (35). Please see Figure 4 for a flowchart of OCS donor heart preservation process. Finally, device malfunction, although uncommon, remains possible (38).

The PROCEED II trial demonstrated noninferiority of OCS relative to SCS with respect to short-and intermediate-term survival, non-fatal major adverse cardiac events, and freedom from cardiac allograft vasculopathy (CAV), although statistical significance has not been consistently demonstrated. The trial established comparable one-year survival between modalities, although several grafts were discarded due to rising lactate despite coronary flow optimization as well as technical issues. Five hearts in the OCS group were discarded (of 67 in the OCS group) due to underlying anatomic or structural issues that could have affected function. Reasons for discard included a history of cardiac arrest complicated by myocardial hemorrhage and necrosis, myocardial scarring from cocaine use, LVH overlooked during procurement, congenital aortic valve fusion causing regurgitation and unstable perfusion pressure, and aortic friability from a connective tissue disorder that complicated instrumentation. The investigators, however, were unsure whether these grafts would have failed if transplanted as these dynamic changes would have gone undetected with traditional SCS (35, 39).

The OCS Heart Registry, initiated in 2023, provides contemporary multicenter data on the use of OCS from donors after brain death (DBD) and donors after circulatory death (DCD) in comparison to the use of SCS. Early analyses of 3,225 transplants across 56 U.S. centers demonstrated that OCS-preserved grafts (which often travel substantially longer distances and include a higher proportion of DCD and extended criteria donors) achieve survival outcomes comparable to SCS-preserved DBD grafts, although statistical significance has not been consistently demonstrated (37).

Thus far, research indicates that one year survival rates are equivalent to SCS (37). Meta-analyses further confirm noninferiority of OCS to SCS for early and late term survival (40), and no significant difference between the two modalities in rejection rates or freedom from chronic allograft vasculopathy (CAV) (35, 39).

An emerging application of normothermic ex-vivo perfusion is its role in facilitating “beating heart” transplantation. In this approach, the donor heart is maintained in a continuously perfused, metabolically active state from procurement through implantation. This technique still entails an initial warm cardioplegia injection after the donor aorta is cross clamped and just prior to the cardiectomy, before the heart is subsequently instrumented into the OCS circuit. However, it forgoes the routine second cardioplegia administration of cold solution during recipient implantation. The benefit to this is the avoidance of exposure to an additional round of cardioplegia by maintaining continuous coronary flow, which eliminates additional ischemic time prior to reperfusion. Consequences mitigated by avoiding more ischemia include myocardial stunning, endothelial injury, inflammatory activation, and cellular damage. This is particularly pertinent in DCD donors, as these hearts have already experienced significant warm ischemic times between the donor's circulatory arrest and OCS cannulation. Early evidence has propelled this line of reasoning, with a small case series finding that 0% of 10 patients receiving the beating heart technique required postoperative ECMO while 40% of 50 patients receiving standard DCD implantations with SCS required postoperative ECMO. This beating heart paradigm is preliminary but represents a conceptual shift from traditional ischemia-based preservation toward continuous organ support. Early clinical experience suggests that this strategy may improve graft utilization rates, although long-term outcomes and standardized protocols remain under investigation (35–37, 41–43).

## Hypothermic oxygenated perfusion

**Figure 5:**
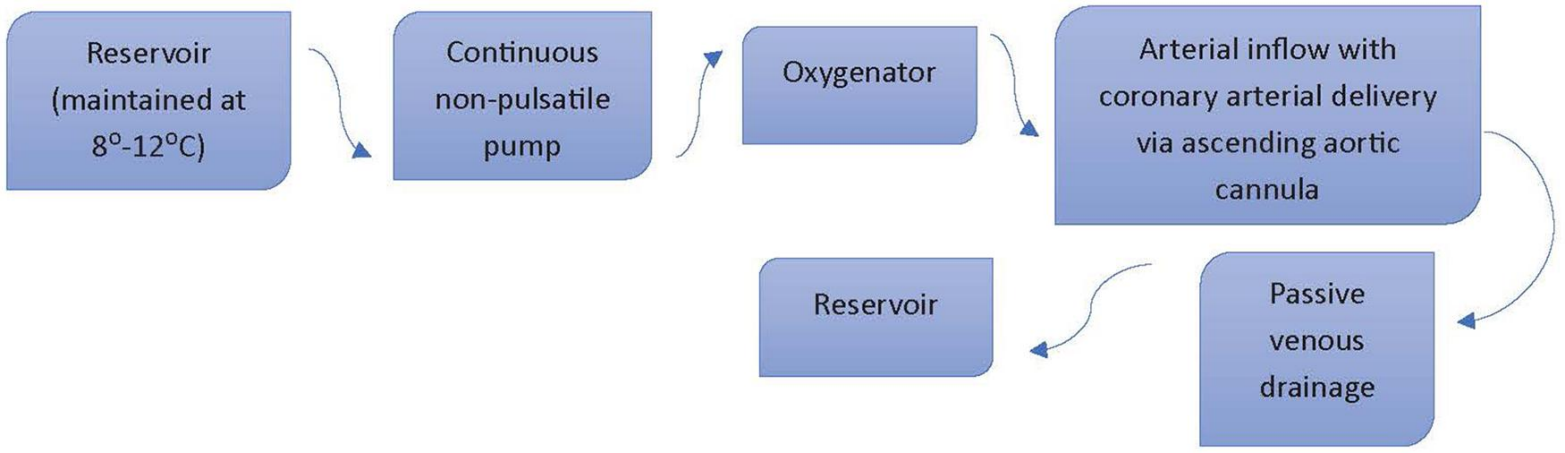
Flow through the HOPE machine.

Hypothermic oxygenated perfusion (HOPE) represents a hybrid approach that combines the metabolic suppression of hypothermia with active oxygen delivery. In this system, oxygenated perfusate is continuously delivered to the coronary arteries of the donor heart at low temperatures (typically 8 degrees C) in a non-pulsatile manner (19). Please see Figure 5 for a schematic of flow through the HOPE machine.

The hypothermia distinguishes it from OCS, and the application of perfusion distinguishes it from SCTS. HOPE provides the most active mitigation of IRI of all these modalities. It has been shown to provide safe preservation for transport times up to nine hours (in stark contrast to the strong recommended limit of four hours that SCS provides) (44). HOPE is also suggested to preserve mitochondrial and endothelial function (19).

A study by Rega et al. demonstrated a marked reduction in severe PGD among HOPE-preserved grafts compared with SCS as well as a 44% risk reduction in a composite primary outcome including PGD, cardiac death, graft failure, and rejection, although statistical significance was narrowly missed. Importantly, all grafts in this trial survived 30 days despite prolonged transport times (19).

**Figure 6:**
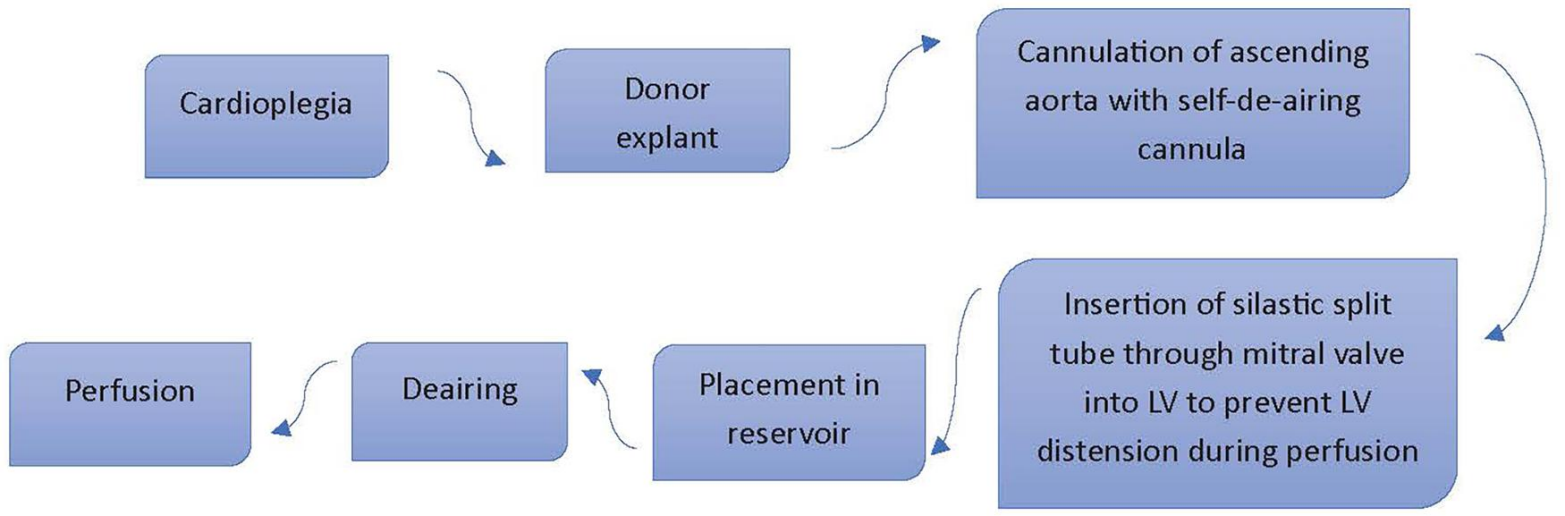
Process from donation to transplant in HOPE.

HOPE systems are technically simpler than normothermic perfusion platforms (which include restoration of normal sinus rhythm among other facets) and carry fewer manipulation risks, though aortic cannulation and circuit connection remain critical steps. Please see Figure 6 for a flowchart of OCS donor heart preservation process. Device-related issues during priming have been reported, occasionally necessitating conversion to SCS. While early function and histologic outcomes appear favorable, long-term data regarding survival, rejection, and CAV remain limited due to relatively recent clinical adoption (19, 35).

## Protocol-level considerations in cardiac allograft preservation

While these preservation techniques are commonly discussed at the device level, there are important protocol-level considerations between them that impact patient outcomes and modality implementation. Considerations include variations in perfusion pressure, perfusion temperature, perfusate composition and volume, and the degree to which these techniques are tailored to the risk profiles of both donors and recipients. In normothermic ex-vivo machine circulatory systems like OCS, the donor heart is kept at nearly physiologic temperature (34 °C) with continuous pulsatile perfusion. Perfusion pressure goals are approximately 60–90 mmHg for mean aortic pressure (the normal physiologic range), coronary blood flow of 650–850 mL/min, and a heart rate of 65–100 beats per minute when pacing is required. To accomplish this, various infusions have been used to control heart rate and vascular resistance, including low-dose adenosine and epinephrine. This allows coronary flow and myocardial metabolism to more closely mimic conditions *in vivo*. Perfusate is generally oxygenated donor blood that is supplemented with nutrients to provide metabolic support. The perfusate is also sampled to assess lactate, and hemodynamic pressure monitoring is performed throughout the circuit (35–37, 45).

HOPE differs from OCS by using substantially lower temperatures (approximately 8° C) with non-pulsatile perfusion and lower perfusion pressures. This balances metabolic suppression with active delivery of oxygen and seeks to preserve mitochondrial integrity via energy supply while minimizing IRI. A perfusion pressure of 20mmHg has been cited in the literature, alongside a pressure-controlled flow of around 150–200 mL/min. The perfusate generally differs in composition from normothermic systems, consisting of oxygenated crystalloid or an oxygenated albumin-based hyper-oncotic solution with meropenem and added hormones rather than whole blood (19, 44).

Systems such as SCTS use strict temperature regulation without providing active support via perfusion. Temperature is maintained in a narrow range (4–8 °C), to reduce the risks of hypothermia and hyperthermia to the graft. Perfusion parameters are not applicable here, but protocol-level differences include an extended viable period of preservation, and enhanced temperature consistency throughout the storage period in comparison to SCS. The cooling mechanism of SCTS is based on a specialty phase-change material which has unique properties that allow the storage and release of large amounts of energy (26, 27).

Traditional SCS is entirely passive. After cardioplegic arrest, the heart is stored in a hypothermic environment without any support. Ideal temperature for hypothermic storage is estimated to be around 4–8°C based on animal models, but temperature monitoring is not routinely performed in SCS. Animal studies have shown that hearts stored on ice reach 1°C in about one hour, and this is of particular concern since excessive cooling (especially below 2°C) is associated with greater risk of cold-induced graft injury. Since the main modifiable variable is preservation time, this is where focus is directed when utilizing SCS. Outcomes are strongly linked to CIT, with CIT periods greater than 3–4 h posing considerably increasing risks of IRI. Accordingly, SCS protocols are highly sensitive to logistics including transport distance and coordination between the procurement team, the transport service, and the donation team (2, 27).

It is key to understand that preservation strategies may change based on anticipated ischemic time and the risk profile of the donor. For standard-risk donors with expected CIT under 3–4 h, SCS remains ubiquitous. However, prolonged transport times, extended criteria donors, or DCD donors frequently spur the usage of advanced preservation techniques such as OCS or HOPE. Both allow metabolic support while OCS has the added benefit of viability assessment to ensure the quality of these at-risk donor grafts. Preservation strategies are not only dependent on the device itself but must be chosen appropriately to each clinical context. Please see Table 1 for a side-by-side comparison of donor heart preservation technologies.

**Table 1:** Comparison of donor heart preservation technologies.

Name	Static Cold Storage (SCS)	Paragonix SherpaPak^TM^ Cardiac Transport System (SCTS)	TransMedics Organ Care System^TM^ (OCS)	Hypothermic Oxygenated Perfusion (HOPE)
Mechanism	Hypothermic metabolic suppression	Controlled hypothermic metabolic suppression	Normothermic *ex vivo* oxygenated perfusion	Hypothermic *ex vivo* oxygenated perfusion
Temperature	Variable. Ideally 4°–8°C	4°–8°C, tightly regulated	34°C	∼8°C
Oxygenation/Perfusion	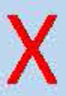	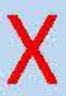	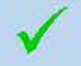 Yes (pulsatile)	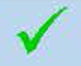 Yes (non-pulsatile)
Viability Assessment	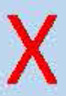	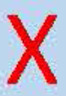	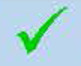 Lactate, flow, pressure, contractility	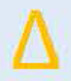 Limited (less than OCS)
Typical Transport Window	Under 3–4 h (ideally)	∼5 h clinically (up to 30 h theoretical) (25)	∼6–9 h, (longest clinical time was about 9 h) (34)	Has been shown safe up to 9 h (40)
PGD/ Outcomes	∼ 29% increased risk of mortality per hour >3 h CIT (tang et al.) (20)	4.0% less severe PGD vs. SCS (33) 3.8% less RV dysfunction (33) vs. SCS	Non inferior survival vs. SCS (34) Enables DCD use (36)	44% risk reduction of composite including severe PGD vs. SCS (19)
Advantages	✓ Simple ✓ Low cost ✓ Widely Available	✓ Precise temperature control ✓ Reduced IRI vs. SCS ✓ Easy adoption	✓ Viability testing ✓ Expands donor pool ✓ Less IRI ✓ Allows for beating heart transplantation	✓ Strong IRI mitigation ✓ Mitochondrial protection
Limitations	✗ No oxygen/Metabolic support ✗ Time- dependent IRI	✗ No perfusion or assessment	✗ Complex logistics ✗ Cannulation risks ✗ Expensive	✗ Limited data ✗ Complex setup with cannulation risks ✗ Likely expensive ✗ Not approved yet
Cost	Minimal (“ice + cooler”)	∼ $17,000 direct equipment cost (26)	∼ $40,000 (26)	Likely high (not published)
Best Use Scenario	Standard-risk, short distance, pediatric	Prolonged CIT	DCD, extended-criteria donors, marginal grafts	Extended-criteria donors, prolonged CIT, DCD
Key Evidence	UNOS registry (40,052 patients) With normal CIT ∼91% 1 year survival (22)	GUARDIAN registry (33) EMB IRI reduction study (31)	PROCEED II trial (34) OCS Registry (3,225 pts) (36)	RCT (rega et al., Lancet 2024) (19)

## Donation after circulatory death and normothermic regional perfusion

DCD has emerged as a major strategy for expanding the heart donor pool. Early data suggest that short and long-term outcomes including survival, severe PGD, rejection, and CAV are comparable to those seen with donation after brain death (DBD). The challenge unique to DCD, however, is the obligatory warm ischemia interval that follows withdrawal of life-sustaining therapy. As such, the graft must be reanimated and assessed before it can be reimplanted. There are two principal recovery techniques that have been adopted. The first is direct procurement and perfusion, in which the heart is explanted and reanimated on an *ex vivo* normothermic perfusion platform (as described with OCS). The other is thoracoabdominal normothermic regional perfusion (TA-NRP), in which the heart is reanimated *in situ* by restoring warm, oxygenated blood flow via an extracorporeal circuit after ligation of the arch vessels to prevent cerebral reperfusion.

TA-NRP has several practical advantages. It allows functional graft assessment via echocardiography and hemodynamic monitoring prior to procurement. Additionally, it interrupts warm ischemia earlier and facilitates simultaneous abdominal organ recovery. Despite favorable outcomes, TA-NRP adoption has been constrained by ethical debate over whether restoration of circulation to the donor's body after circulatory death violates the dead donor rule, even with arch vessel ligation in place. A scoping review of 112 publications found no consensus on the ethical permissibility of NRP. TA-NRP remains prohibited in several countries and restricted at a large percentage of US hospitals and organ procurement organizations. Ongoing empirical studies to definitively establish absence of brain blood flow during TA-NRP is critical to enable broader adoption (46–48).

## Preservation solutions in SCS

Preservation solutions remain a cornerstone of SCS and continue to influence outcomes. In SCS, the donor heart is flushed with and then submerged within a cold cardioplegic preservation solution that serves to reduce metabolic demands by maintaining hypothermia and cardioplegia. These solutions also aim to reduce cellular edema and calcium overload during cold storage by slowing the rate of ATP depletion to maintain the function of ion pumps amongst other metabolic functions. Commonly used solutions include the University of Wisconsin (UW) solution (ViaSpan®, Bridge to Life Ltd., Columbia, SC, USA), Celsior® solution (Institut Georges Lopez, Lissieu, France), and Custodiol Histidine-Tryptophan-Ketoglutarate (HTK) solution (Custodiol®, Dr. Franz Köhler Chemie GmbH, Bensheim, Germany) Histidine-Tryptophan-Ketoglutarate (HTK). These solutions differ in chemical composition and intracellular vs. extracellular design, with UW and HTK formulated as intracellular (high potassium and low sodium) and Celsior as extracellular (low potassium and high sodium) (49, 50). Generally, intracellular solutions have <70 mEq/L of sodium (mimicking intracellular fluid), while extracellular solutions have ≥70 mEq/L of sodium (like extracellular fluid) (50). Intracellular fluids work by slowing the rate of damaging cellular edema. When the Na+/K+ ATPase pump fails, sodium accumulates intracellularly with water following it. Solutions with low intracellular sodium help to minimize the osmotic gradient drawing water into the cells (51). These solutions also commonly include impermeant compounds that cannot cross cell membranes, high energy phosphate precursors to provide building blocks for ATP generation on reperfusion, and antioxidants to mitigate free radical injury at reperfusion (52). Clinical analysis indicates that intracellular solutions are associated with decreased one-month mortality compared to extracellular solutions (50). Extracellular solutions focus on calcium homeostasis. The myocardium is unique amongst organs in benefiting from controlled calcium presence alongside magnesium to prevent calcium flux. This prevents the “calcium paradox” of reperfusion causing calcium influx and hyper contracture (53). Experimental data have suggested that extracellular solutions provide better cardiac functional recovery than intracellular solutions, and this is hypothesized to be due to the presence of calcium (54). These solutions may also provide antioxidants (55), glutamate, and other ATP substrates (53).

Studies in both adult and pediatric populations suggest either noninferior or superior outcomes when using UW vs. either Celsior or HTK solutions. One meta-analysis found that UW is associated with improved 30-day and 90-day survival and reduced ischemic necrosis (on early biopsy samples) compared with Celsior (49). Higher necrosis scores have been associated with increased PGD and right heart failure (56). A UNOS analysis additionally suggested that UW is associated with improved short term (30 day and one year) survival vs. Celsior, particularly in high-risk allografts characterized by older donor age or extended ischemia (57). A best evidence review advocates for the use of UW in SCS, especially in extended ischemia times or when utilizing marginal donors. This is based on the rationale that intracellular solutions like UW may offer better short-term survival than extracellular solutions like Celsior (58). However, Celsior has largely been found effective in SCS despite being associated with slightly worse outcomes than UW (59). A large UNOS study for pediatric heart transplant found no significant difference in 30 day or 1 year survival between Celsior and UW, suggesting that differences in outcome may be less relevant in pediatric or lower risk populations (60). Experimental studies thus far suggest that Celsior may be less effective in protecting against IRI in older donor hearts or in prolonged cold ischemia, while HTK hearts show lower markers of injury and better functional recovery vs. Celsior (61, 62). HTK itself appears to show clinically safe and effective outcomes in clinical use, with low rates of severe PGD and high survival rates (63).

Collectively, these findings indicate that all three solutions are clinically acceptable. Solution selection should be considered in conjunction with preservation modality and donor risk profile rather than in isolation. Please see Table 2 for a side-by-side comparison of donor heart preservation solutions.

**Table 2:** Comparison of common donor heart preservation solutions.

Solution	University of Wisconsin/ViaSpan^TM^ (UW)	Celsior^TM^	Custodiol^TM^ HTK
Type	Intracellular (high K^+^; low Na^+^)	Extracellular (low K^+^; high Na^+^)	Intracellular (high K^+^; low Na^+^)
Key Composition Features	Lactobionate, raffinose (impermeants) (44) Adenosine (ATP precursor) (44) Glutathione (antioxidant) (44)	Glutamate (45) Mannitol (45) Magnesium (45)	Histidine buffer (41) Trytophan (41) Ketoglutarate (41)
Mechanism	Prevents cellular edema Maintains ATP stores	Calcium homeostasis Reduces oxidative stress	Strong buffering capacity Supports metabolism on reperfusion
Advantages	✓ ischemic necrosis (41) ✓ Better short- term survival (41)	✓ Good functional recovery (53)	✓ Low injury markers (53) ✓ Good functional recovery (53)
Limitations	✗ High viscosity ✗ Less calcium buffering	✗ ↑ ischemic injury vs. UW (41)	✗ Dilutional effects (large volume)
Clinical Evidence	Meta-analysis: ↓ 30- and 90- day mortality (41) UNOS survival advantage (49)	Slightly worse outcomes vs. UW (41, 49)	Safe clinical outcomes (55) Experimental superiority vs. Celsior (53)
Best Use	High-risk grafts Prolonged CIT	Standard-risk grafts	Prolonged ischemia Older donor hearts

## Controversies and considerations

As preservation strategies have evolved beyond static cold storage, the field has entered an era in which multiple technically feasible approaches exist, each with distinct biological, logistical, and economic tradeoffs. This expansion has shifted debate away from whether donor hearts can be preserved safely, toward how preservation should be optimized and when advanced modalities can meaningfully improve outcomes.

One prevailing school of thought maintains that static cold storage (SCS) should remain the default preservation strategy for most heart transplants. Proponents emphasize its simplicity, low cost, established safety profile, broad global applicability, and minimal requirement for graft manipulation. Importantly, outcomes with SCS remain excellent in standard-risk settings (particularly when CIT is limited), and in pediatric transplantation (19). From this perspective, routine replacement of SCS is neither necessary nor sufficiently supported by long-term outcome data. Instead, advanced preservation technologies are considered best reserved for select scenarios, such as anticipated prolonged ischemia time (>3–4 h), extended transport distances, or with the use of extended-criteria donors (64).

In contrast, proponents of advanced preservation modalities argue that machine-based approaches actively mitigate IRI and therefore address the biological limitations of SCS (65). These technologies may expand the donor pool, improve utilization of marginal grafts, and reduce early complications such as PGD. Reliance on SCS alone risks constraining geographic allocation and perpetuating avoidable graft discard, particularly as broader donor criteria are increasingly adopted.

Despite these potential advantages, concerns regarding advanced preservation technologies persist. These include limited long-term outcome data, increased logistical complexity, the need for specialized personnel, and substantially higher upfront costs. SCS is consistently described as inexpensive in the literature, but data on exact costs are not described. This method may be termed “ice and a cooler” and generally only requires sterile ice, preservation solution, packaging, and a cooler (2). In contrast, more advanced modalities have much higher operating costs and expertise requirements. The literature on pricing is sparse, but normothermic *ex vivo* perfusion systems such as OCS have been estimated at tens of thousands of dollars in up-front cost per use, with one source citing a figure of $40,000 (27). SCTS poses a more intermediate option, with the same source quoting it at $17,000 (27). The cost of HOPE is not described in the literature nor on the website of the main commercial provider. However, the provider does state online that they offer funding for clinical trials and industry sponsored research grants (66). As a machine perfusion technology, it likely costs more than SCTS and almost certainly substantially more than SCS due to the logistical and technical complexities involved in operation. Such differences in pricing, logistics, and requisite specialized skills raise important questions regarding cost-effectiveness, scalability, and equitable access, particularly in healthcare systems with constrained resources. It makes sense that SCS as a “set it and forget it” technique offers benefits in terms of simplicity and ease of access over systems requiring more intensive manipulation and monitoring.

Notably, cost considerations may not be linear. Controlled hypothermic storage platforms, while more expensive than traditional SCS, can have potential downstream cost savings in specific contexts. By reducing rates of severe primary graft dysfunction, intensive care utilization, and post-transplant mechanical circulatory support, these systems may offset initial expenditures and, in some settings, yield net institutional savings. These findings highlight the complexity of evaluating preservation strategies solely on acquisition cost and underscore the importance of considering total episode-of-care expenditures.

Taken together, these perspectives reflect a central tension in modern heart transplantation: balancing biological optimization with practical feasibility and economic sustainability. Rather than supporting a single universal approach, preservation strategy selection should be individualized. It should incorporate donor and recipient risk profiles, anticipated ischemia duration, institutional expertise, and resource availability. Ongoing prospective studies and longer-term comparative data will be essential to determine whether advanced preservation technologies should complement or ultimately redefine the current standard of care.

## Current research gaps

Despite substantial innovation in cardiac allograft preservation, important knowledge gaps remain that limit the development of evidence-based, widely generalizable preservation strategies. These gaps span biologic mechanisms, clinical implementation, and study design. They are particularly relevant as advanced preservation technologies move from selective use toward broader adoption.

Further investigation into adjunctive pharmacological and mechanical cardioprotective interventions during preservation is needed. Additionally, topics such as donor-recipient size matching and the influence of pre-transplant recipient management (including mechanical circulatory support, inotrope exposure, and end-organ dysfunction) on susceptibility to IRI and post-transplant outcomes remains incompletely characterized.

From a clinical and methodological standpoint, the lack of standardized protocols across preservation technologies represents a major barrier to meaningful comparison. Variability in implementation introduces substantial confounding into observational studies and limits the interpretability of existing data. Standardization is further needed for graft assessment protocols during perfusion, preservation solution composition and dosing, and definitions of extended donor acceptance. Without consensus on these elements, outcomes across studies remain difficult to compare and generalize.

Finally, there is a notable absence of large-scale, multicenter randomized controlled trials directly comparing preservation modalities and solutions across diverse donor and recipient populations. Outcomes are often reported in broad ischemic time intervals without integration of transport logistics, device-specific variables, or clinically meaningful endpoints such as severe primary graft dysfunction or post-transplant resource utilization. Addressing these limitations will require study designs that incorporate multidimensional risk stratification and standardized reporting frameworks.

Collectively, closing these research gaps will be essential to inform the development of evidence-based guidelines that move beyond a one-size-fits-all approach. Integrating mechanistic insights with rigorously standardized clinical data has the potential to refine preservation strategies, optimize donor organ utilization, and improve outcomes across the increasingly heterogeneous heart transplant population.

## Potential developments in the field

Heart transplantation continues to evolve and undergo constant innovation, research, and improvement. Preservation solutions are increasingly being refined to include cardioprotective agents that further optimize post-storage viability (67). Precision preservation technologies that utilize biologically driven perfusates (such as HOPE) and targeted pharmacological agents (e.g., valproic acid) are being explored (68).

Furthermore, biomarker-driven graft assessment represents a rapidly advancing area of investigation. Technologies such as cell-free DNA analysis, gene expression profiling, and real-time perfusate biomarker analysis offers the potential for objective, dynamic assessment of viability during preservation. Integration of these tools, however, remain largely investigational, and further validation is needed before clinical adoption (69, 70).

Artificial intelligence (AI) and machine learning (ML) represent a natural extension of these biomarker-driven approaches and are increasingly being explored across all phases of organ transplantation. In the pre-transplant setting, ML algorithms have been explored to optimize donor-recipient matching by integrating a variety of datasets including clinical, demographic, and immunological variables. AI analytics hold promise for integrating real-time perfusate biomarker data, hemodynamic monitoring, and donor characteristics during perfusion to generate dynamic assessments of graft variability. This could play an important role in guiding individualized preservation strategies and supporting clinical decision-making regarding graft acceptance and rejection. Furthermore, AI tools show promise in assisting with personalized immunosuppression regimens by predicting individual patient responses based on clinical data. There are significant barriers, however, to clinical use due to the need for large-scale validation, data standardization across transplant centers, algorithmic transparency, and regulatory approval. Multicenter studies will be essential to develop and further grow AI methods that are generalizable and applicable across different transplant populations.

Machine perfusion for organ preservation continues to undergo refinement. As described above, this is aiding in extending viable ischemia times, reducing IRI, allowing real-time lab sampling for active assessment, and expanding the pool of donor organs by allowing harvest from extended criteria donors. These platforms are also being integrated with molecular and functional biomarker interpretation to customize treatment. Applications of this include early detection of graft injury, personalized risk stratification, and individualized immunosuppression regimens (71, 72). On a macroscopic level, global synchronization of regulatory frameworks, training programs, and device availability is a crucial next step in standardizing high quality care and expanding access to advanced technologies. This must be tempered with realistic plans for locations with less economic means by using best evidence to maximize outcomes and value.

Beyond preservation, parallel research into xenotransplantation, organ reconditioning, and tissue engineering continues to advance, offering the potential for transformative expansion of the donor pool. While these approaches remain largely experimental, their convergence with advances in preservation science underscores a broader shift toward biologically integrated solutions to organ scarcity (73). Along similar lines, the rapid recovery with extended ultraoxygenated preservation (REUP) technique is a new and novel method that challenges the pre-existing concept that DCD hearts must be reanimated prior to implantation. REUP delivers an oxygenated perfusate as an extended infusion over 10–12 min after which the heart is transported in standard cold storage. There is no *in situ* reperfusion, no machine perfusion platform, or any specialized perfusion team. If and once validated in larger studies, REUP and similar strategies could make DCD heart procurement even more accessible (74, 75).

Based on current evidence, several actionable recommendations exist. For standard-risk transplants with anticipated short ischemic intervals (<4 h), particularly in pediatric patients, SCS remains an effective and widely applicable strategy, with acceptable outcomes using commonly employed preservation solutions. In adult recipients or high-risk scenarios (such as older donor age or ischemia exceeding 4 h), the UW solution appears to confer advantages in early outcomes.

Advanced preservation modalities, including controlled hypothermic storage, normothermic *ex vivo* perfusion, and hypothermic oxygenated perfusion, are most appropriately considered in settings involving extended criteria donors, prolonged transport times, or long-distance procurement. When machine perfusion platforms are employed, perfusate analysis for graft injury (extracellular vesicles, injury markers, oxidative stress) is recommended (76).

Finally, while prevention of early PGD through mitigation of IRI remains a central goal, long term graft survival is primarily determined by the incidence of complications such as chronic rejection and CAV (77). Effective management therefore requires not only optimized preservation but also durable immunosuppression, structured and personalized surveillance protocols based on risk (possibly including coronary angiography every 6–12 months), and aggressive modification of cardiovascular risk factors (78).

## Conclusion

Advances in heart transplantation have transformed survival for patients with end-stage heart failure, yet donor organ scarcity and preservation-related graft injury remain central constraints on further progress. While SCS has served as the foundation of cardiac allograft preservation for decades and continues to deliver excellent outcomes in standard-risk settings, its inherent limitations (most notably time-dependent IRI) have become increasingly apparent as donor criteria expand, and allocation networks broaden.

The emergence of controlled hypothermic storage systems, normothermic *ex vivo* perfusion, and hypothermic oxygenated perfusion reflects a broader shift toward biologically informed preservation strategies. These modalities aim not only to extend preservation time but also to actively mitigate ischemic injury, improve early graft function, and enable safer utilization of extended criteria and geographically distant donors. Available evidence suggests that no single approach is universally superior; rather, each modality offers distinct advantages and tradeoffs that must be weighed against donor risk, recipient characteristics, transport logistics, institutional expertise, and resource availability.

Current controversies in the field underscore the need for individualized preservation strategies rather than wholesale replacement of established standards. SCS remains appropriate for many transplants, particularly those involving short ischemic intervals and pediatric recipients, while advanced modalities appear most beneficial in higher-risk scenarios. Importantly, economic considerations, logistical complexity, and access disparities must be integrated into preservation decision-making alongside biological outcomes.

Looking forward, the greatest opportunities for progress lie in closing existing research gaps through standardized protocols, robust multicenter randomized trials, and long-term outcome reporting. Integration of molecular and functional biomarkers into preservation platforms holds promise for dynamic graft assessment and personalized management but requires further validation. At the same time, regulatory frameworks and scalable implementation strategies will be essential to ensure that innovation translates into equitable improvements in care.

Ultimately, optimization of heart transplant preservation should be viewed as a continuum rather than a binary choice between modalities. By aligning preservation strategy with donor and recipient risk profiles, advancing mechanistic understanding of IRI, and moving forward through rigorous clinical evidence, the field is well positioned to expand the donor pool, reduce early graft dysfunction, and improve long-term outcomes for an increasingly diverse transplant population.

## Clinical application and decision-making framework

Based on current evidence, preservation strategy selection can be approached using a practical framework incorporating donor risk, anticipated ischemic time, and institutional capabilities.

**Figure 7:**
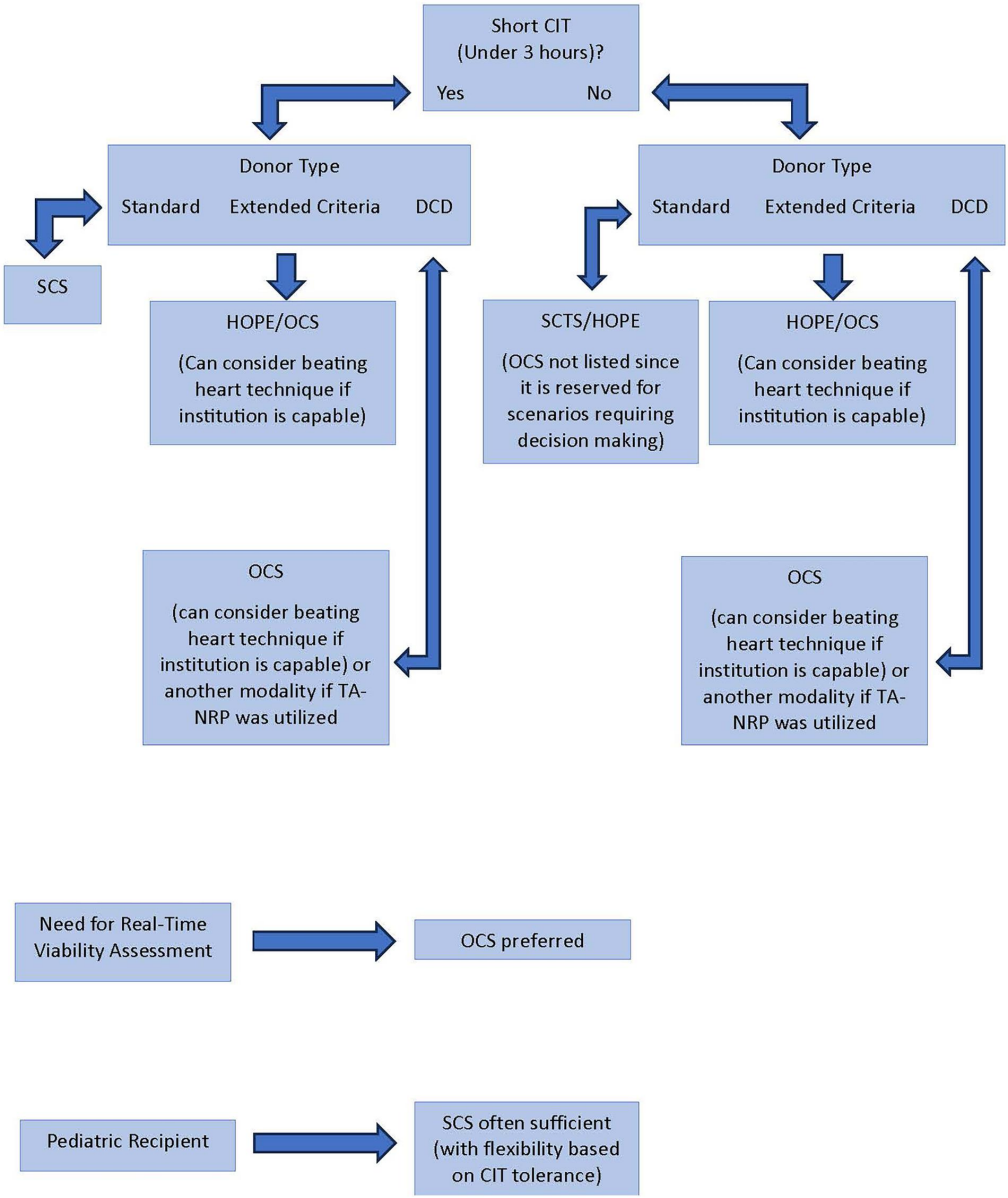
Decision making flowchart: which modality to use?

For standard-risk adult donors with anticipated cold ischemia time under approximately 3–4 h, static cold storage remains an effective and widely accessible strategy with excellent outcomes. In cases involving prolonged ischemic time, extended transport distance, or higher-risk donor characteristics (such as older donor age or marginal graft quality), advanced preservation modalities should be considered. Controlled hypothermic storage systems such as SCTS may offer improved temperature stability and reduced ischemic injury while maintaining logistical simplicity. When real-time graft assessment is desired, particularly in the setting of DCD or marginal donors, normothermic *ex vivo* perfusion systems such as OCS provide the advantage of continuous metabolic support and viability assessment. HOPE represents an alternative approach that combines oxygen delivery with metabolic suppression and may be particularly beneficial in scenarios with prolonged ischemia times and grafts in need of support. Pediatric heart transplantation represents a distinct clinical context, as available evidence suggests greater tolerance to prolonged ischemic time compared with adults. As such, SCS remains appropriate in many pediatric cases, although advanced modalities may still be considered in selected high-risk situations. Please see Figure 7 for a decision tree flowchart on optimal heart preservation modality selection.

Ultimately, preservation strategy selection should be individualized. It should integrate donor and recipient characteristics, anticipated ischemic duration, need for viability assessment, transport logistics, and institutional expertise.
