# Supplementary material for: Contemporary strategies for donor heart preservation in heart transplantation
Source: Front Transplant. 2026 Jul 2;5:1825471. doi: 10.3389/frtra.2026.1825471 (PMC13373086; doi:10.3389/frtra.2026.1825471)
Supplement: Supplementary file 1 [file Supplementaryfile1.docx]

**PLEASE NOTE:** Images references appear at the end of this supplemental file. Our proprietary images are indicated. Each photo number is listed with associated reference.

**Photos:**

SCS- PHOTO 1, Our proprietary image


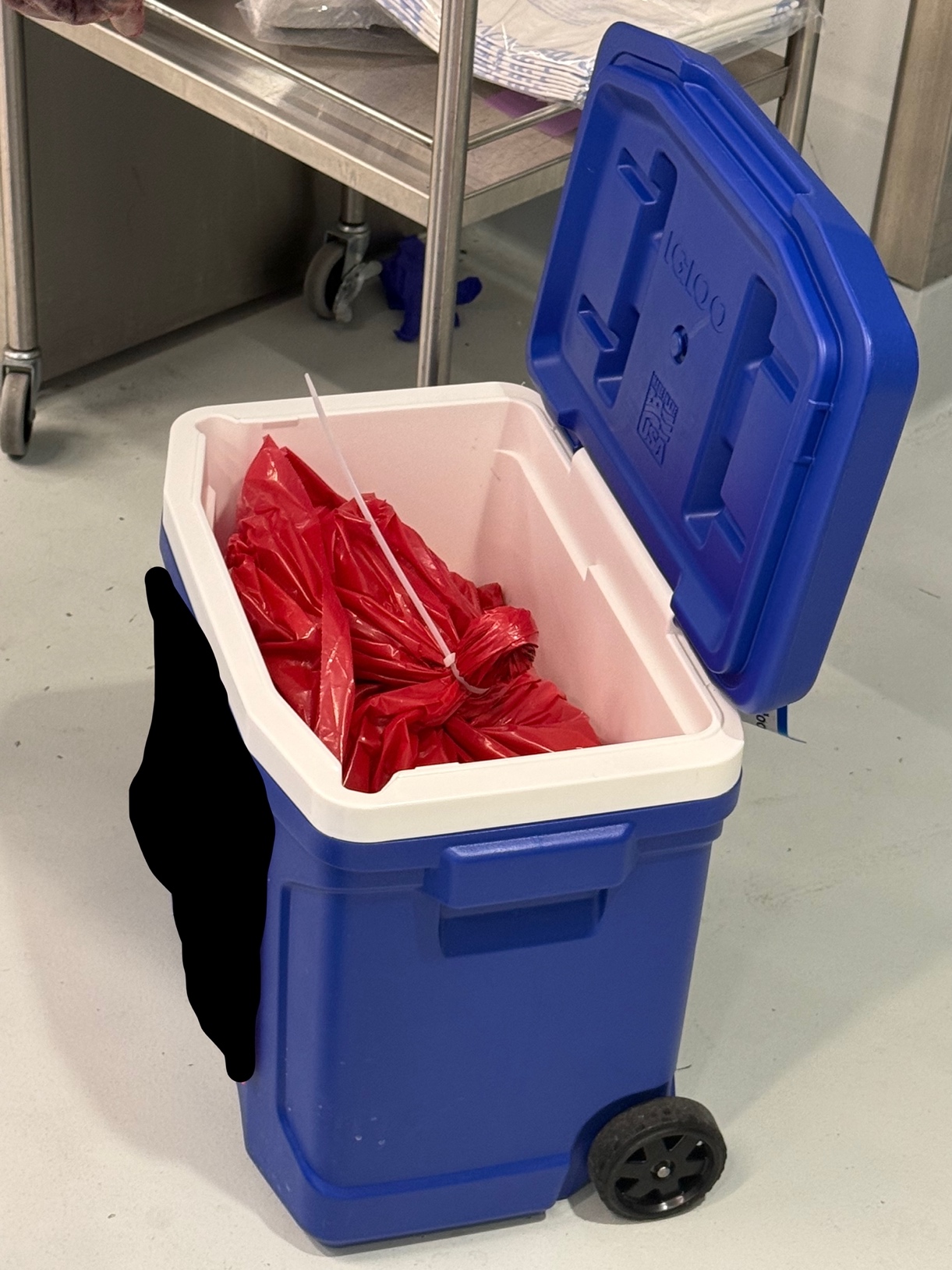


Heart within SCS system in a cooler.

SCS- PHOTO 2, Our proprietary image


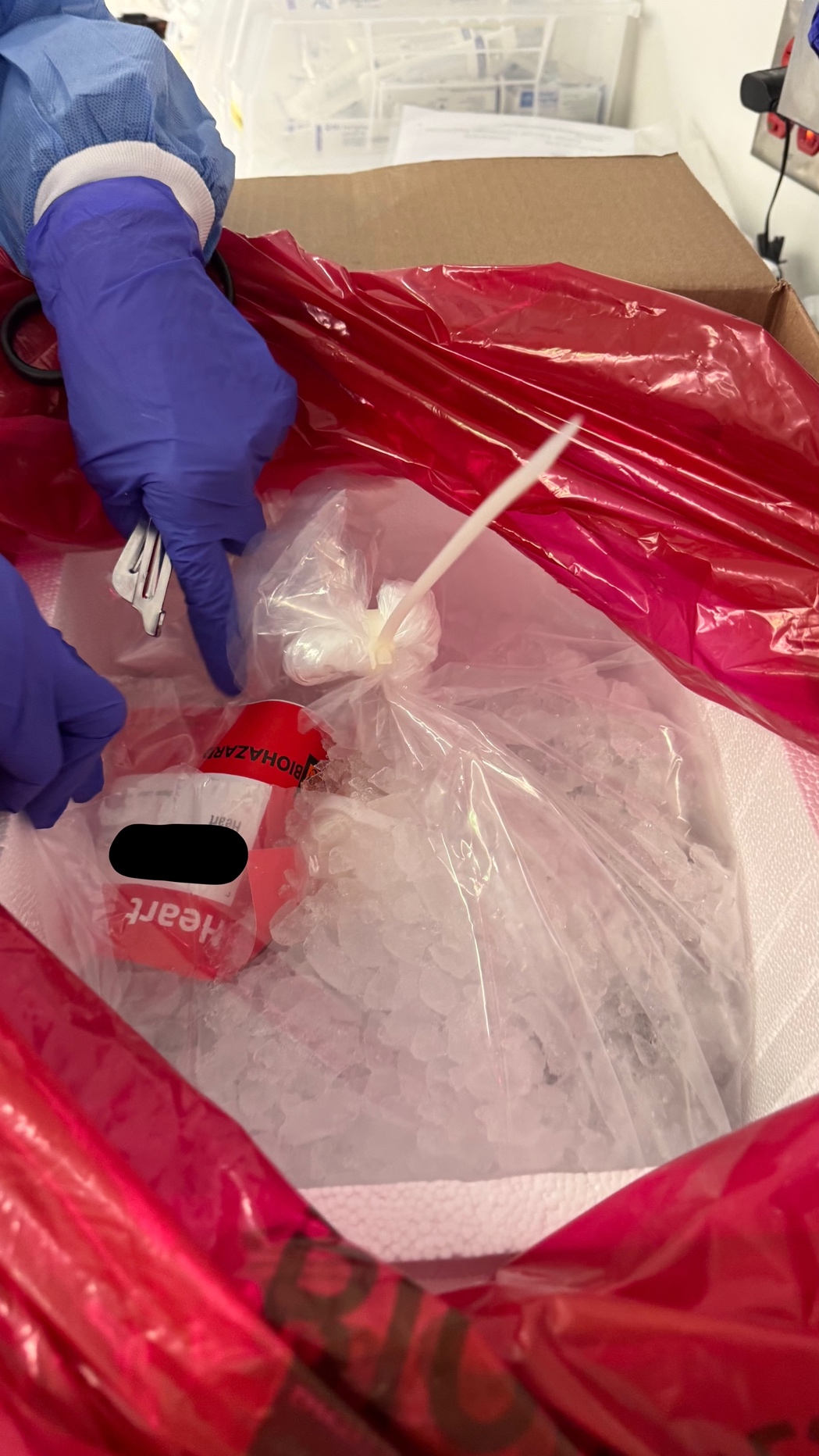


Opening of the heart cooler.

SCS- PHOTO 3, Our proprietary image


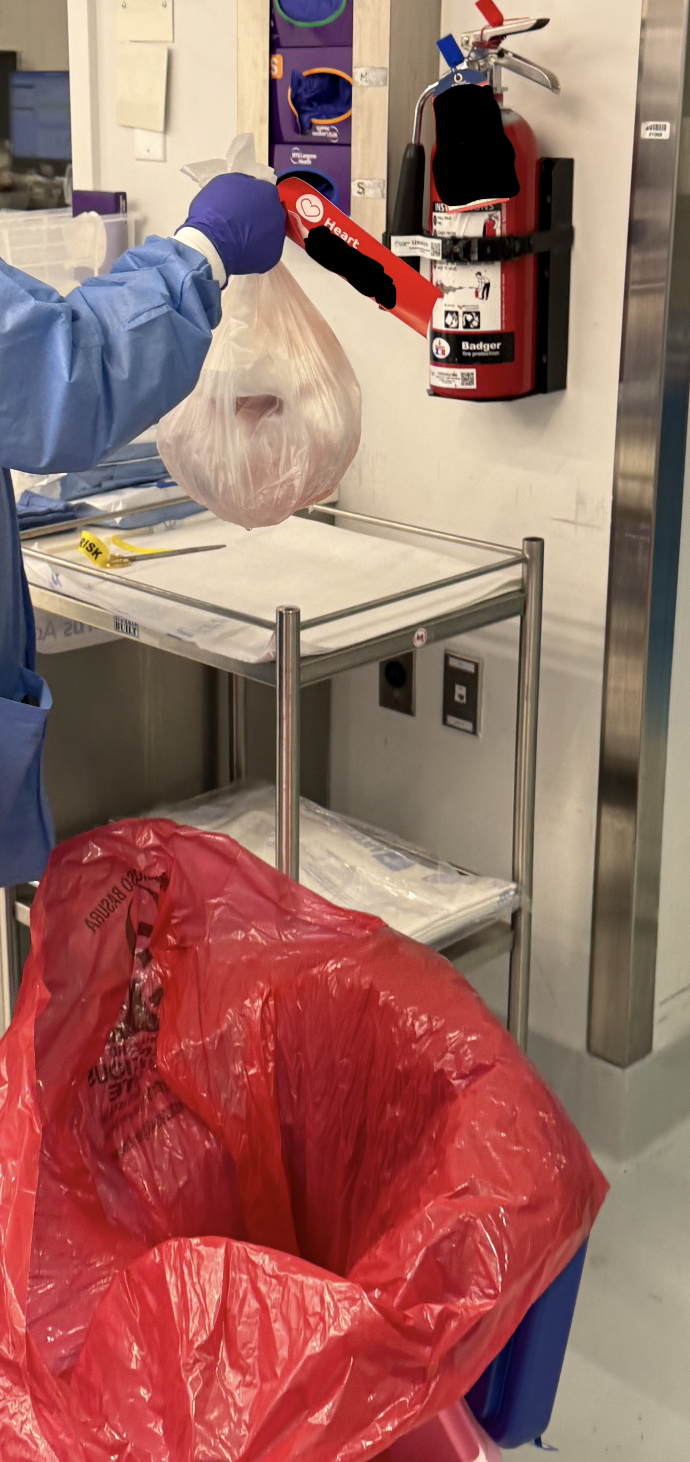


Removal of bagged heart from SCS cooler.

SCS- PHOTO 4, Our proprietary image


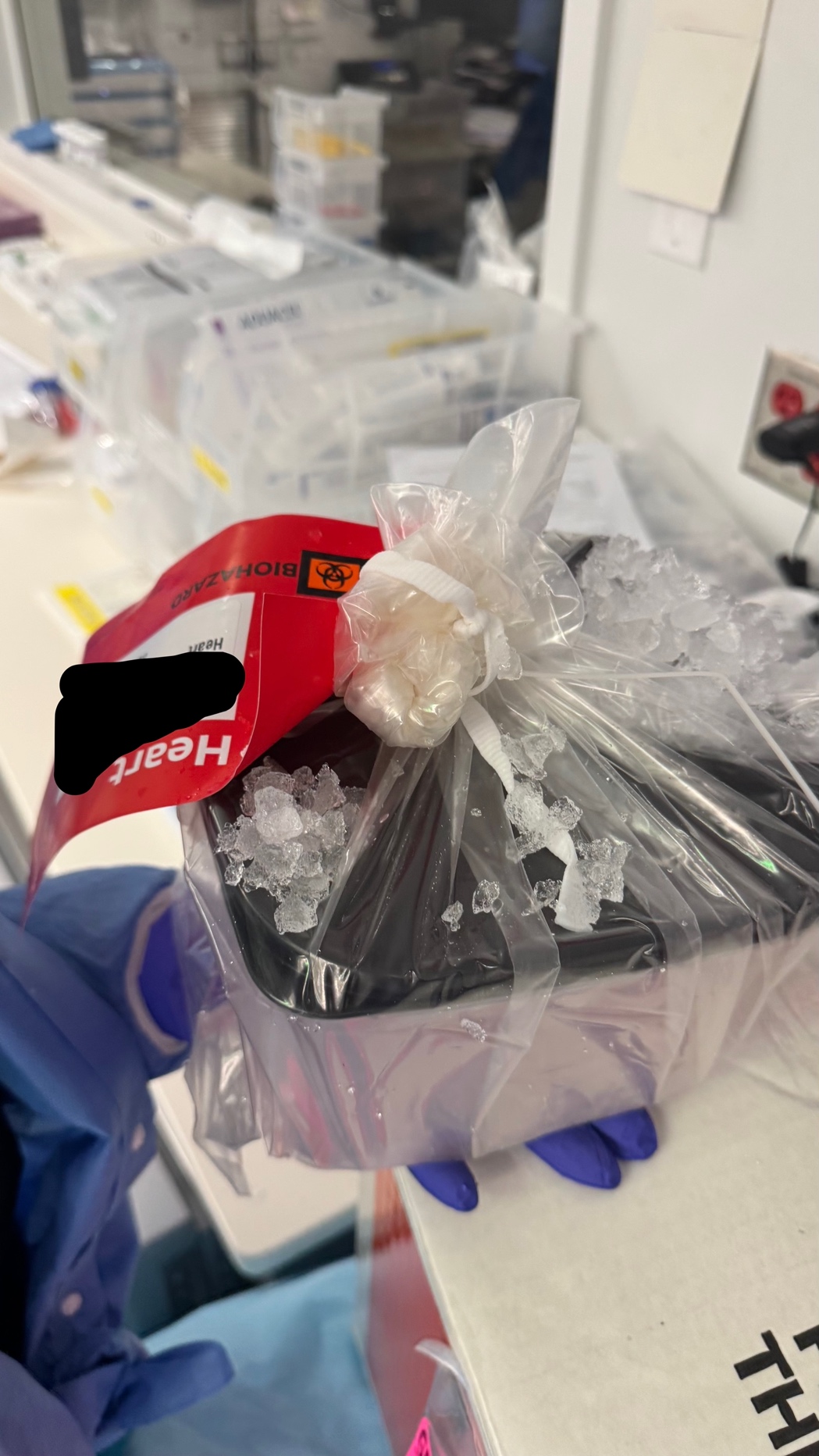


Further unbagging of the heart preserved within the SCS system.

SCS- PHOTO 5, Our proprietary image


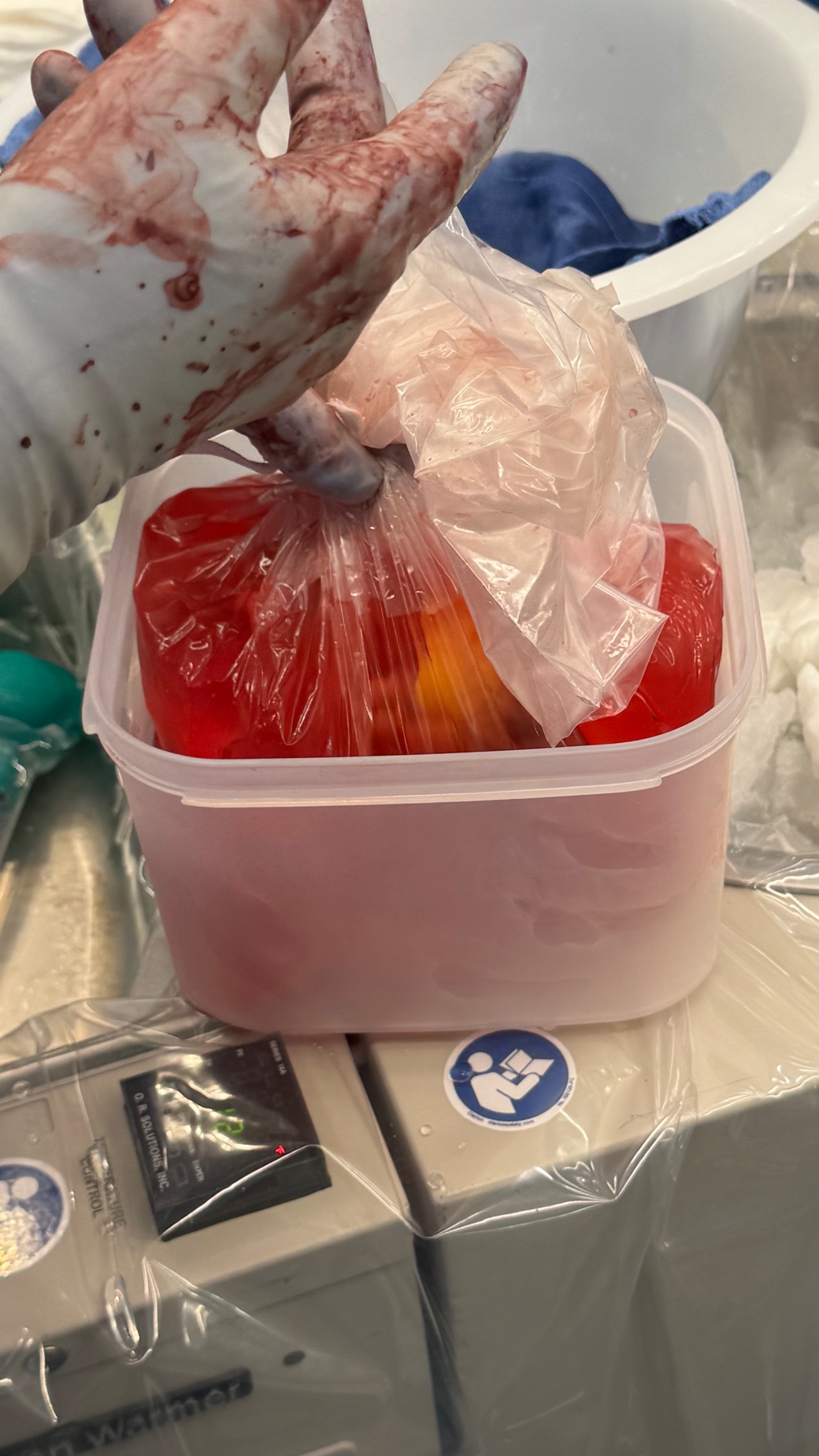


The inner bag of the SCS system.

SCS- PHOTO 6, Our proprietary image


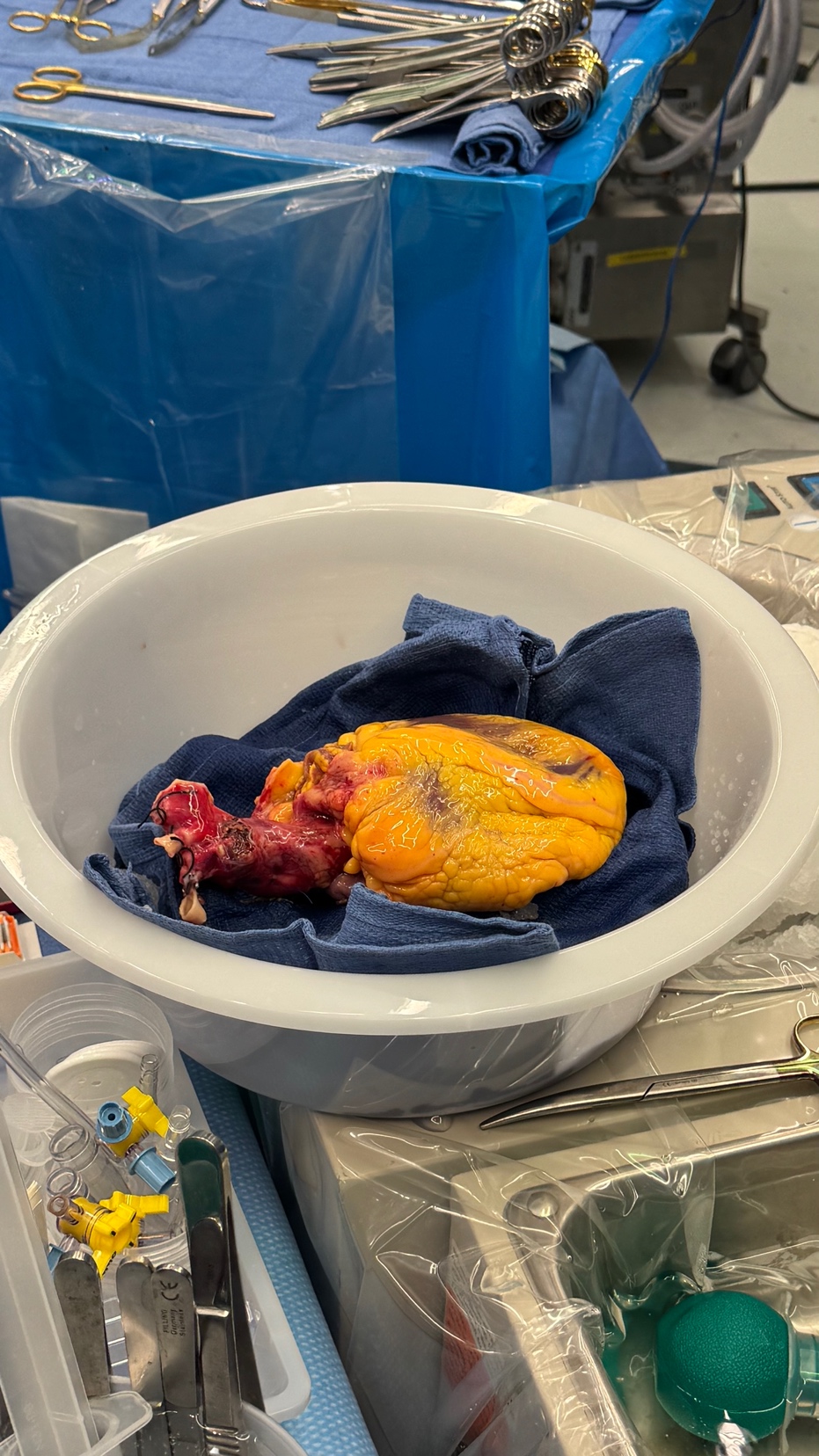
 The heart after being completely removed from SCS system.

SCTS - PHOTO 7, Reference 79


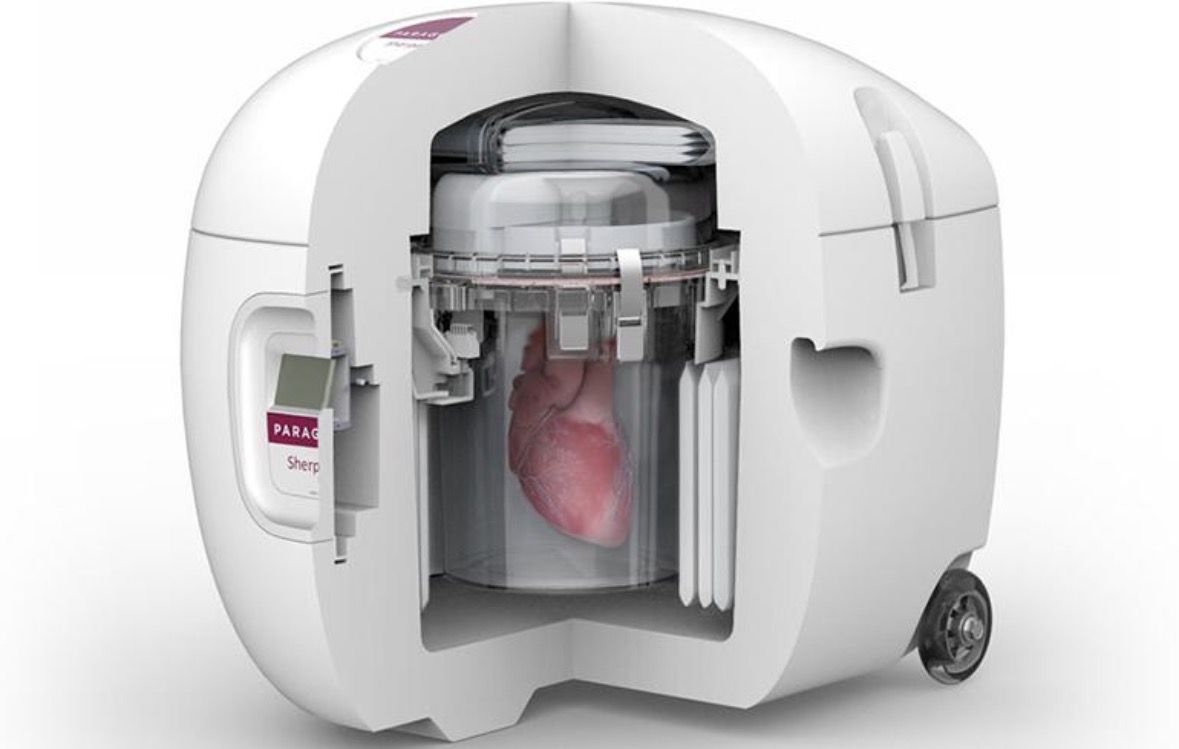


The SCTS module.

PHOTO 8, Reference 79


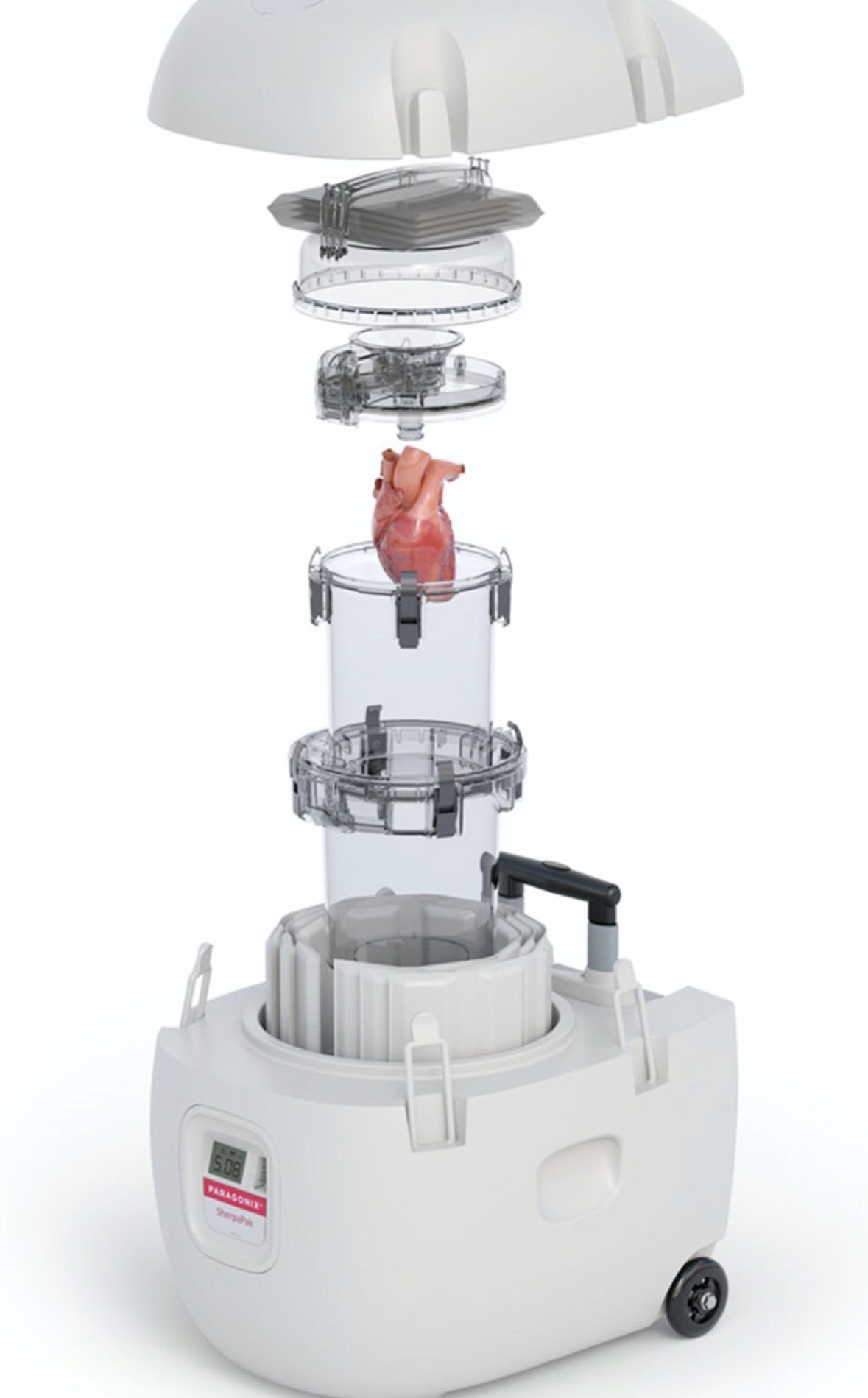


A cross-sectional view of the SCTS system.

HOPE - PHOTO 9, Reference 80


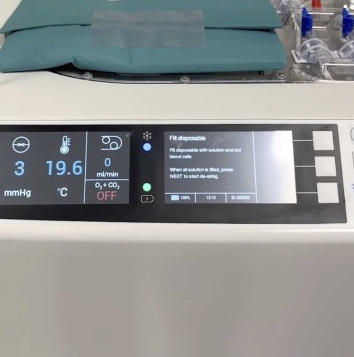


The HOPE machine display.

PHOTO 10, Reference 80


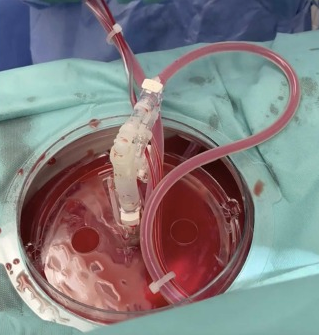


A heart within the HOPE machine.

Labelled Diagrams:

OCS - PHOTO 11, Reference 81


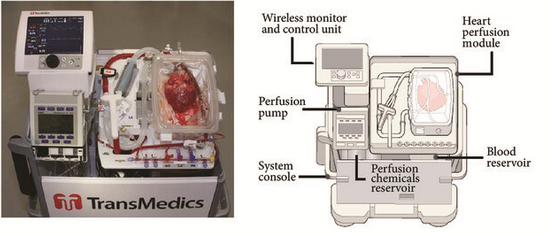


The OCS perfusion circuit and monitor (left), a diagram with the labelled components (right).

HOPE

PHOTO 12, Reference 82


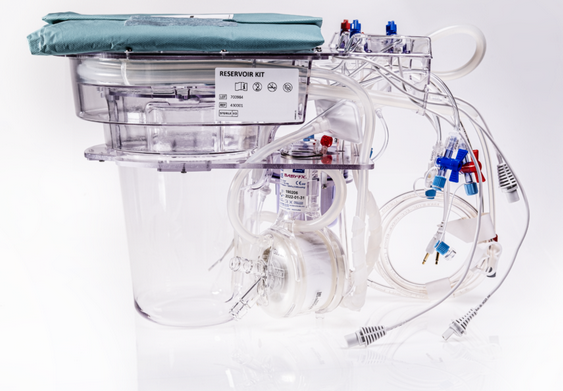


The inner circuitry of the HOPE machine. As it is not commercially available and does not have regulatory approval in any market, images are sparse.

**Flow diagrams:**

**SCS**

**Process from donation to transplant:**

Aortic cross clamp → Cardioplegia → Donor explant → Cold immersion in 3 bag system (2–8°C) → Transport on ice in cooler→ Implantation

**SCTS**

**Process from donation to transplant:**

Aortic cross clamp → Cardioplegia → Donor explant → Placement in inner cannister → Controlled 4–8°C environment → Monitored transport → Implantation

OCS - PHOTO 13, Reference 79


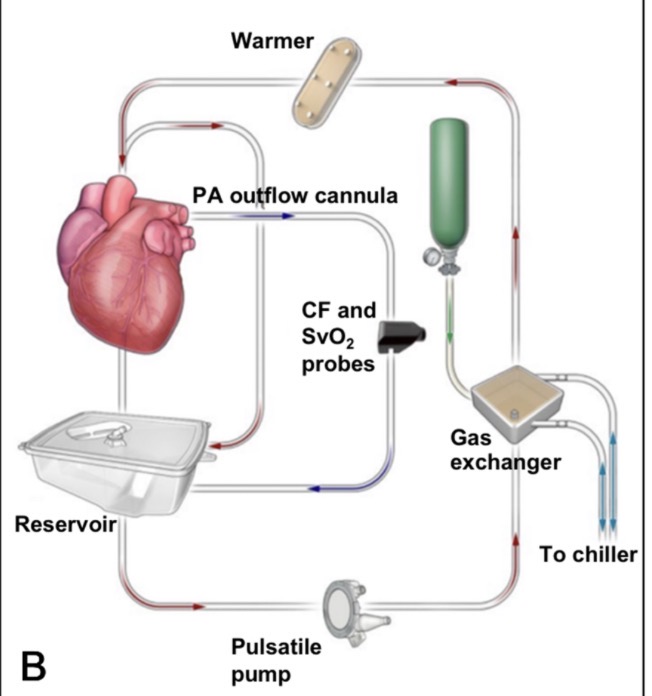


**Flow through the OCS machine:**

Reservoir → pump → oxygenator → warmer → aorta → coronary perfusion → coronary sinus flow through tricuspid valve (SVC and IVC are closed) → ejection by RV into PA cannula → passage through coronary flow/SvO2 sensors → Reservoir → recirculation

**Process from donation to transplant in OCS:**

Warm cardioplegia → Donor explant → Cannulation → Connection to OCS circuit → Warm pulsatile oxygenated perfusion and reanimation→ Continuous monitoring (lactate, flow) → Transport → Cold cardioplegia (unless beating heart implantation) → Implantation

HOPE

**Flow through the HOPE machine:**

Reservoir (maintained at 8-12 degrees C) → Continuous non-pulsatile pump → Oxygenator → Arterial inflow with coronary arterial delivery via ascending aortic cannula→ Passive venous drainage → Reservoir

**Process from donation to circuit incorporation in HOPE:**

Cardioplegia → Donor explant → Cannulation of ascending aorta with self-de-airing cannula → Insertion of silastic split tube through mitral valve into LV to prevent LV distension during perfusion → Placement in reservoir → Deairing → Perfusion

**References**

79. Leon, M. (2024). Revolutionizing Donor Heart Procurement: Innovations and Future Directions for Enhanced Transplantation Outcomes. Journal of Cardiovascular Development and Disease, 11(8), 235. Retrieved from https://www.mdpi.com/2308-3425/11/8/235

80. Ngeve, S., Kucera, J. A., Wolf, S. E. M., Aykut, B., Omer, T. M., Murillo-Berlioz, A., et al. (2025). First US pediatric heart transplant using XVIVO Heart Assist Transport. JHLT Open, 10. doi:10.1016/j.jhlto.2025.100381

81. Sunjaya, A. F., & Sunjaya, A. P. (2019). Combating Donor Organ Shortage: Organ Care System Prolonging Organ Storage Time and Improving the Outcome of Heart Transplantations. Cardiovascular Therapeutics, 2019(1), 9482797. doi:https://doi.org/10.1155/2019/9482797

82. Transport, X. H. A. (2026). XVIVO Perfusion AB. Retrieved from https://www.xvivogroup.com/products-services/xvivo-heart-assist-transport/

Photos:

Photos 1-6: Proprietary photos taken by Dr. Israeli and associate authors.

Photo 7, 8, 13: Reference [79]

Photo 9-10: Reference [80]

Photo 11: Reference [81]

Photo 12: Reference [82]
